# Drug-Induced Hyponatremia Surveillance: A disproportionality analysis based on FAERs database

**DOI:** 10.12669/pjms.42.4.13918

**Published:** 2026-04

**Authors:** Xin Xi, Zhanfeng Bai, Songqing Liu, Jie Dong

**Affiliations:** 1 Xin Xi Pharmacy Department, The Third Affiliated Hospital of, Chongqing Medical University, Chongqing, China; 2 Zhanfeng Bai Pharmacy Department, The Third Affiliated Hospital of, Chongqing Medical University, Chongqing, China; 3 Songqing Liu Pharmacy Department, The Third Affiliated Hospital of, Chongqing Medical University, Chongqing, China; 4 Jie Dong Pharmacy Department, The Third Affiliated Hospital of, Chongqing Medical University, Chongqing, China

**Keywords:** FAERS database, Hyponatremia, Reporting odds ratio

## Abstract

**Objective:**

Mining the risk signals of drug-induced hyponatremia by the United States Food and Drug Administration adverse event reporting system (FAERS) database, to provide reference for clinical safe medication.

**Methodology:**

Adverse drug events related to drug induced hyponatremia were analyzed by disproportionality analysis using the FAERS data between 2004 to 2023 were downloaded (https://fis.fda.gov/extensions/FPD-QDE-FAERS/FPD-QDE-FAERS.html).

**Results:**

A total of 1,306 drugs with identifiable risk signals were associated with Hyponatremia. Five drugs demonstrated the highest cases counts: furosemide (n=1,560), hydrochlorothiazide (n=1049), sertraline (n=899), omeprazole(n=864), citalopram (n=738). The pharmacovigilance analysis identified five substances exhibiting the strongest association signals: indapamide (ROR=91.46, 95% CI 83.38-100.32), hydrochlorothiazide (ROR=46.79, 95% CI 43.96-49.81), desmopressin (ROR=34.08, 95% CI 31.49-36.89), oxcarbazepine (ROR=24.47, 95% CI 22.72-26.37), and furosemide (ROR=24.11, 95% CI 22.91-25.37). Nivolumab (36.92%), bortezomib (30.58%), and zoledronic (29.36%) exhibited the highest proportional occurrence of target adverse events involving fatal or life-threatening outcomes. Additionally, clinical information about Drug-induced hyponatremia is absent from the Summary of Product Characteristics of 10 drugs in our study.

**Conclusions:**

We provide a list of drugs with risk signals for Hyponatremia. In clinical practice, it is helpful to identify the culprit drug early, and prevent it from further aggravation into a serious condition.

## INTRODUCTION

Hyponatremia (serum Na <135 mmol/L) is the most prevalent electrolyte disorder in hospitalized patients, linked to longer hospital stays and readmission.^1^ Its clinical severity depends on both the degree and rate of serum Na decline: mild-moderate cases (125-134 mmol/L) have nonspecific symptoms (often masked by comorbidities), while serum Na <125 mmol/L raises neurological sequelae (cognitive dysfunction, gait instability) risk and increases mortality by 55%.^2^,^3^ Early detection and targeted management are thus critical for high-risk groups.

Drugs are a definitive cause of hyponatremia, via impaired renal Na regulation and dysregulated antidiuretic hormone (ADH) secretion. Syndrome of inappropriate ADH secretion (SIADH) accounts for 60% of hyponatremia cases; medications cause 18% of SIADH.^4^,^5^ SIADH is strongly associated with psychotropics (antipsychotics, antidepressants), anticonvulsants, antineoplastics, and emerging culprits like proton pump inhibitors (PPIs), calcium channel blockers (CCB), and renin-angiotensin-aldosterone system inhibitors (RAASi).^6^

While drug-induced hyponatremia gains more clinical recognition, current studies mainly focus on discrete drug classes, with marked interstudy heterogeneity in reported incidence. For example, hyponatremia incidence is 0.06%-40% in selective serotonin reuptake inhibitors (SSRIs) users, 4.8%-41.5% in antiepileptic drug users; antitumor therapy patients have rates >40%, possibly due to comorbidities.^4^,^5^,^7^ Additionally, small-sample studies and case reports suggest potential links between hyponatremia and drugs like PPIs, immunoglobulins, CCBs, and angiotensin II receptor antagonists (ARBs)^8^,^9^ but systematic data on their incidence and risk stratification remains sparse. Moreover, comparative analyses of hyponatremia risk across drug classes and polypharmacy effects remain uncomprehensive in existing literature.

This study leverages FDA Adverse Event Reporting System (FAERS) database to identify hyponatremia risks across medications, identifying high-incidence agents and establishing clinical decision-support parameters for safer prescribing practices, while constructing an epidemiological foundation for investigating drug-induced sodium homeostasis disruptions.

## METHODOLOGY

FAERS quarterly data (ASCII format) from January 2004 to December 2023 were downloaded (https://fis.fda.gov/extensions/FPD-QDE-FAERS/FPD-QDE-FAERS.html). To minimize confounding by other drugs, only reports listing the target drug as the “primary suspect” were included. Duplicate reports were removed using the FDA’s preferred method: for each CASEID, the report with the maximum FDA_DT was retained; and for identical CASEID and FDA_DT, the report with the highest PRIMARYID was kept. Cases of hyponatremia were identified using the MedDRA Preferred Term (PT) “HYPONATREMIA” (code 10021036). Drug names were standardized to generic names and coded according to the WHO ATC classification. Data mining was performed using R software (v4.3.1).

### Ethical statement;

As FAERS data is anonymized, no ethical assessment was required.

### Disproportionality analysis:

The reporting odds ratio (ROR) was used for disproportionality analysis to detect potential adverse drug event (ADE) signals by comparing the proportion of a target event for a drug of interest against that for all other drugs. The RORs for the drugs that caused Hyponatremia were calculated by using a two-by-two contingency table, as shown in Table-I. ROR=ad/bc, and 95% confidence interval (CI)=e^ln(ROR)±1.96(1/a+1/b+1/c+1/d)^0.5^. When a≥3 and the lower end of the 95% CI for the ROR value is higher than One, the potential risk signal of ADE is satisfied. To enhance accuracy, analysis was performed using ASCII data from the FDA approval date of each drug through the fourth quarter of 2023.

**Table-I:** Two-by-two contingency table for disproportionality analysis.

	Target ADEs	Other ADEs	Total
Target drugs	a	b	a+b
Other drugs	c	d	c+d
Total	a+c	b+d	n=a+b+c+d

ADE: adverse drug event; a: the number of cases with the target ADE of the target drug; b: the number of cases with all other ADEs of the target drug; c: the number of cases with the target ADE of all other drugs; d: the number of cases with all other ADEs of all other drugs.

### Time-to-onset (TTO) analysis:

TTO was defined as the interval between the AE date (EVENT_DT) and drug start date (START_DT). Data with erroneous or missing dates were excluded. TTO was summarized using medians, interquartile ranges (IQR), and Weibull’s shape parameter (WSP). The Weibull distribution, characterized by scale (α) and shape (β) parameters, identified three failure types: early failure (β < 1, hazard decreases over time), random failure (β is equal to or close to One, constant hazard), and wear-out failure (β > 1, hazard increases over time), based on the β value and its 95% confidence interval.

## RESULTS

### Descriptive analysis:

**Fig.1:**
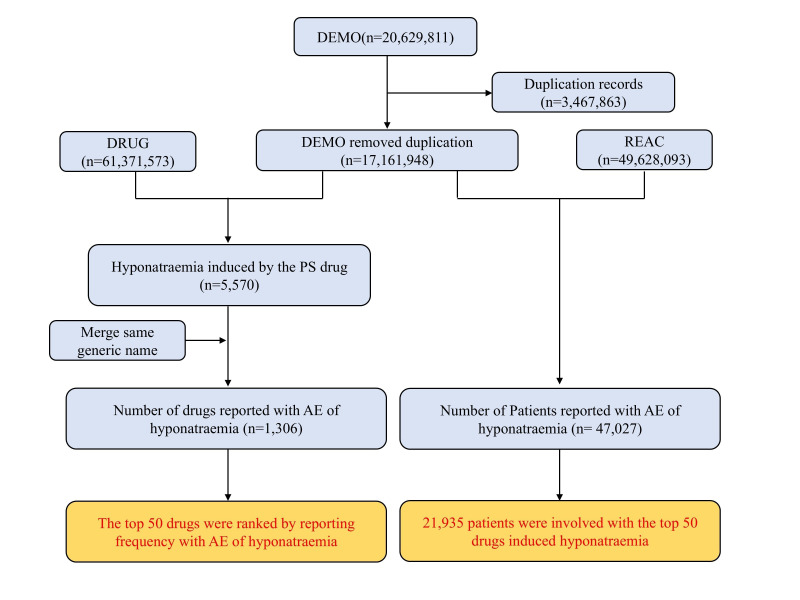
Flow chart for identification cases of Hyponatremia in our study. PS, primary suspected; ADE, Adverse Drug Event.

**Fig.2:**
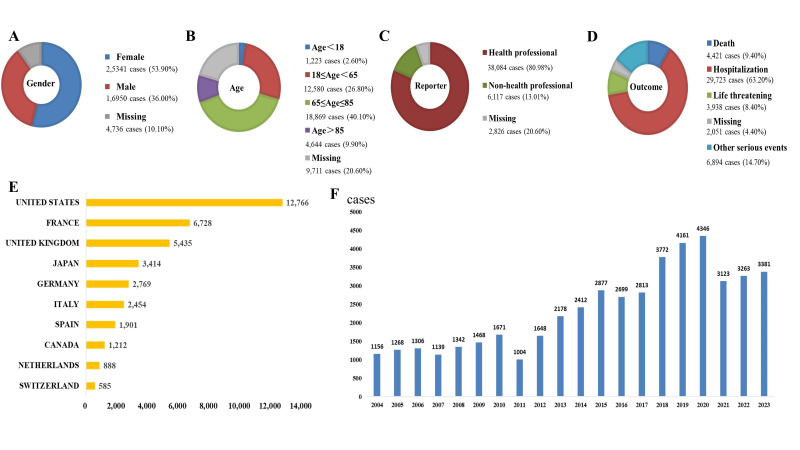
Demographic characteristics and reporting information for the top 50 drugs with AE of Hyponatremia. (A) Gender distribution, (B) Age decile stratification, (C) Reporter qualification categories, (D) Clinical outcome severity, (E) Country-specific reporting frequencies, (F) Case volume temporal trends.

A total of 20,629,811 records were extracted from FAERS (Jan 2004–Dec 2023). After removing duplicates, 1,306 drugs were linked to 47,027 hyponatremia patients; the top 50 hyponatremia-linked drugs involved 21,935 patients’ total. Data mining process is shown in Fig.1. Among 47,027 patients, 18,869 (40.10%) were aged 65–85 years; females (n=25,341, 53.90%) outnumbered males (n=16,950, 36.00%). Total 8,359 patients (17.8%) had death or life-threatening outcomes. Additionally, the US had the highest case count (n=12,766) among reporting countries, and healthcare professionals reported more cases than non-healthcare professionals (Fig.2).

### Culprit-drug list induced Hyponatremia:

**Fig.3:**
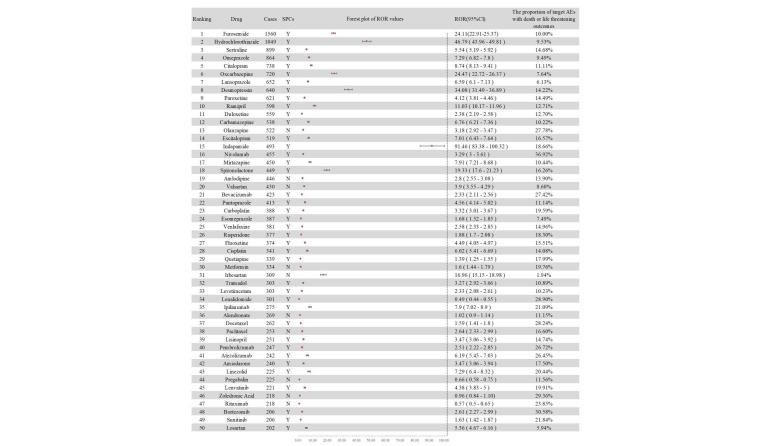
The top 50 drugs associated with ADE of Hyponatraemia.

The top three drugs with the highest number of reported cases were furosemide(1,560 cases), hydrochlorothiazide(1,049 cases), and sertraline(899 cases), and the three drugs with the highest ROR values were indapamide(ROR= 91.46, 95%CI: 83.38 ~100.32), hydrochlorothiazide(ROR= 46.79, 95%CI: 43.96 - 49.81), and desmopressin(ROR=34.08, 95% CI: 31.49~36.89). The top three drugs in the proportion of target AEs with death or life-threatening outcomes were nivolumab (36.92%), bortezomib (30.58%), and zoledronic acid (29.36%). In addition, The SPCs for each of the 50 drugs were reviewed, 10 drugs did not list information in their SPCs about adverse reactions Hyponatremia (Fig.3).

### Time-to-onset analysis:

Except Pregabalin, the upper limits of the 95% confidence intervals for all shape parameters were below one, indicating an early failure type based on Weibull shape parameter (WSP) analysis. Pregabalin, with a shape parameter of 1.08 (95% CI: 0.93–1.23), exhibited a random failure type. The results of the time-to-onset (TTO) and WSP analyses for the top 50 drugs associated with hyponatremia reports are summarized in Table-II.

**Table-II:** TTO and Webibulll distribution for the top 50 drugs associated with hyponatraemia.

SN	Drug names	Cases	TTO(Days)		Webibulll distribution				Failure type
		n	Median	IQR	Scale parameter		Shape parameter		
					α	95% CI	β	95% CI	
1	Furosemide	535	84.00	16.00-337.50	190.9	160.23-221.57	0.56	0.52-0.59	Early Failure
2	Sertraline	407	10.00	5.00-29.00	36.96	29.33-44.59	0.50	0.47-0.53	Early Failure
3	Hydrochlorothiazide	345	144.00	22.00-757.00	374.36	291.19-457.52	0.50	0.46-0.54	Early Failure
4	Nivolumab	313	47.00	14.00-127.00	82.99	70.56-95.42	0.78	0.72-0.85	Early Failure
5	Citalopram	266	16.00	8.00-117.00	84.36	61.47-107.25	0.47	0.43-0.51	Early Failure
6	Paroxetine	252	9.50	4.00-68.25	52.82	37.07-68.58	0.44	0.40-0.48	Early Failure
7	Oxcarbazepine	234	79.00	15.00-387.00	220.13	166.12-274.14	0.55	0.50-0.61	Early Failure
8	Valsartan	209	41.00	13.00-112.00	77.31	61.99-92.62	0.72	0.65-0.80	Early Failure
9	Ipilimumab	202	60.00	29.50-93.75	94.57	78.16-110.98	0.84	0.76-0.92	Early Failure
10	Irbesartan	198	27.00	10.25-99.25	86.97	64.53-109.40	0.57	0.52-0.63	Early Failure
11	Remipril	195	265.00	90.50-634.50	430.62	347.06-514.17	0.76	0.68-0.85	Early Failure
12	Omeprazole	185	148.00	15.00-1574.00	448.69	315.23-582.15	0.51	0.45-0.57	Early Failure
13	Indapamide	174	16.50	8.25-102.75	73.78	48.86-98.69	0.47	0.42-0.52	Early Failure
14	Duloxetine	172	11.00	4.00-37.75	41.13	27.30-54.97	0.47	0.42-0.52	Early Failure
15	Carboplatin	171	12.00	6.00-37.50	31.56	24.76-38.36	0.74	0.66-0.82	Early Failure
16	Olanzapine	169	13.00	4.00-64.00	53.30	35.36-71.24	0.48	0.42-0.53	Early Failure
17	Carbamazepine	167	55.00	8.50-1011.50	311.66	182.02-441.29	0.39	0.34-0.43	Early Failure
18	Atezolizumab	166	21.00	9.00-62.00	46.82	36.55-57.09	0.74	0.66-0.82	Early Failure
19	Docetaxel	160	8.00	4.75-24.25	22.71	17.25-28.18	0.68	0.61-0.76	Early Failure
20	Escitalopram	153	99.00	15.00-653.00	224.71	151.46-297.97	0.51	0.45-0.58	Early Failure
21	Zoledronic acid	146	72.50	21.25-188.50	132.17	103.64-160.69	0.80	0.70-0.89	Early Failure
22	Pantoprazole	144	16.00	5.00-170.50	88.77	53.57-123.96	0.44	0.39-0.49	Early Failure
23	Paclitaxel	144	14.00	7.00-45.25	32.26	25.52-39.00	0.83	0.73-0.93	Early Failure
24	Desmopressin	143	31.00	5.00-277.00	153.96	85.39-222.53	0.39	0.34-0.44	Early Failure
25	Sunitinib	140	14.00	6.00-39.50	33.10	24.47-41.73	0.67	0.60-0.75	Early Failure
26	Bevacizumab	139	196.00	27.00-847.00	408.68	273.35-544.02	0.53	0.46-0.60	Early Failure
27	Risperidone	130	18.00	10.00-92.00	100.96	62.31-139.61	0.48	0.42-0.54	Early Failure
28	Bortezomib	125	29.00	13.00-84.00	72.94	54.02-91.86	0.72	0.63- 0.81	Early Failure
29	Amlodipine	117	50.00	7.00-133.00	99.99	64.22-135.76	0.54	0.46-0.61	Early Failure
30	Mirtazapine	116	42.00	17.00-259.00	146.48	91.12-201.84	0.51	0.44-0.58	Early Failure
31	Quetiapine	114	51.00	18.50-269.75	142.76	95.48-190.04	0.59	0.50-0.67	Early Failure
32	Pregabalin	108	7.00	4.00-12.00	10.69	8.71-12.67	1.08	0.93-1.23	Random Failure
33	Cisplatin	105	12.00	6.00-28.00	24.87	18.36-31.37	0.78	0.67-0.88	Early Failure
34	Lisinopril	87	40.00	11.00-100.50	65.82	47.07-84.57	0.78	0.65-0.91	Early Failure
35	Rituximab	85	16.00	2.00-195.00	72.71	35.79-109.64	0.44	0.37-0.52	Early Failure
36	Lansoprazole	82	147.00	10.00-1885.00	434.34	206.34-662.34	0.44	0.36-0.51	Early Failure
37	Spironolactone	81	38.00	4.00-178.00	82.63	46.70-118.56	0.53	0.45-0.62	Early Failure
38	Tramadol	79	7.00	4.00-34.00	33.66	16.19-51.13	0.45	0.39-0.52	Early Failure
39	Alendronate	76	240.50	78.75-779.50	445.47	303.81-587.14	0.75	0.61-0.88	Early Failure
40	Venlafaxine	72	58.00	18.00-261.00	179.71	93.39-266.04	0.51	0.42-0.60	Early Failure
41	Linezolid	70	15.00	3.25-64.00	52.24	24.03-80.46	0.46	0.38-0.54	Early Failure
42	Esomeprazole	67	22.00	5.00-148.00	79.70	35.61-123.80	0.46	0.38-0.54	Early Failure
43	Levetiracetam	67	24.00	6.50-303.50	127.84	54.40-201.28	0.44	0.36-0.52	Early Failure
44	Losartan	65	14.00	9.00-73.00	70.99	35.24-106.74	0.51	0.43-0.60	Early Failure
45	Fluoxetine	64	21.50	8.75-430.00	148.41	70.69-226.13	0.50	0.40-0.59	Early Failure
46	Lenvatinib	58	357.00	235.00-1746.00	896.74	528.61-1264.87	0.66	0.53-0.79	Early Failure
47	Pembrolizumab	56	209.50	56.25-616.00	376.55	207.74-545.36	0.62	0.49-0.74	Early Failure
48	Metformin	52	236.00	23.00-1623.00	563.48	301.55-825.41	0.62	0.48-0.75	Early Failure
49	Amiodarone	45	51.00	17.00-338.00	190.69	85.99-295.38	0.56	0.44-0.69	Early Failure
50	Lenalidomide	32	30.00	17.00-185.00	124.71	62.10-187.32	0.73	0.53-0.93	Early Failure

ADE: Adversedrugevent, CI: Confidence interval, IQR: interquartile ranges, SN: serial number, TTO: time-to-onset.

## DISCUSSION

Analysis of drug-induced hyponatremia cases showed 40.1% occurred in patients aged 65-85 years. Existing literature consistently cites advanced age as an independent hyponatremia risk factor, mainly due to age-related reduced renal water excretion. This pathophysiological risk is mainly driven by progressive glomerular filtration rate (GFR) decline, with reduced intrarenal prostaglandin synthesis also involved.^10^ Furthermore, our findings show women (n=25341, 53.9%) have higher hyponatremia risk than men (n=16950, 36%), consistent with established epidemiological patterns.^11^,^12^ This sexual difference seems mediated by falling estrogen/progesterone levels, which boost arginine vasopressin (AVP) secretion via increased hypothalamic osmoreceptor sensitivity, ultimately worsening water retention.^13^

Consistent with prior findings,^6^ diuretics remain the main cause of drug-induced hyponatremia. Our study identified 4 diuretics-furosemide (ROR=24.11, 95%CI 22.91-25.37), hydrochlorothiazide (ROR=46.79, 95%CI 43.96-49.81), indapamide (ROR=91.46, 95%CI 83.38-100.32), spironolactone (ROR=19.33, 95% CI 17.6-21.23)-among the top 50 hyponatremia-linked drugs, all with significant statistical signals.

While clinical studies show thiazide diuretics cause 22.6% (178/786) of severe hyponatremia cases vs. 9.2% (72/786) for loop diuretics,^12^ our FAERS data showed contrasting real-world patterns: furosemide had higher absolute reporting rates (7.11%, 1560/21935) than hydrochlorothiazide (4.78%, 1049/21935). This paradox likely comes from different therapeutic uses: hydrochlorothiazide is used in low-dose as first-line hypertension treatment, while furosemide is preferentially prescribed at higher doses for renal insufficiency/heart failure patients (with inherent sodium homeostasis impairment).^14^

We found indapamide (ROR=91.46, 95%CI 83.38-100.32) and hydrochlorothiazide (ROR=46.76, 95%CI 43.96-49.81) had significantly higher ROR than furosemide (ROR=24.11, 95%CI 22.91-25.37). This difference may be due to thiazide diuretics promoting ADH release (worsening hyponatremia), and indapamide’s longer half-life sustaining these effects.^15^ Prior studies reported variable hyponatremia severity with indapamide;^16^ our study showed it occurs earlier clinically and correlates with higher severe adverse event incidence, suggesting caution in indapamide’s clinical use.

Hyponatremia is the most common electrolyte disorder in cancer patients, with 47% prevalence^17^ and as an independent risk factor for mortality and longer hospitalization. In this study showed 13 of the top 50 hyponatremia-linked drugs were antineoplastics, accounting for 3,737 reported cases. While mechanisms of immune checkpoint inhibitor (ICI)-induced hyponatremia are not fully clear, evidence suggests immune-mediated hypophysitis/adrenalitis causes cortisol deficiency, leading to uninhibited AVP secretion.^18^ One meta-analysis of 6 RCTs showed 9% hyponatremia incidence,^19^ another of 39 RCTs showed 1% (95%CI 0.7%-2.1%).^20^ However, real-world studies show distinct patterns: a single-center retrospective study reported 62% hyponatremia in 2,459 ICI recipients, with 6% severe cases.^21^

Our study further confirms ICI-hyponatremia association: ipilimumab had ROR=7.9 (95%CI 7.02–8.90); nivolumab patients had the highest adverse clinical outcome rate (36.92%), ipilimumab 21.09%. Bevacizumab may increase proteinuria risk (RR=4.79; 95%CI 2.71-8.46) and nephrotic syndrome (RR=7.78; 95%CI 1.80-33.62), thus causing hypervolemic hyponatremia.^22^ Hyponatremia has emerged as its dose-limiting toxicity, with FEARs data indicating adverse clinical outcomes in 27.9% of patients.

Our study shows distinct temporal patterns: sunitinib had a median TTO of 14 days (IQR 6.00-39.50), Lenvatinib 357 days (IQR 235.00-1746.00), showing significant heterogeneity in onset timelines among TKIs. Bortezomib demonstrated clinical relevance in FAERS (ROR=2.61; 95%CI 2.27-2.99), with 30.58% of patients having severe outcomes. Its hyponatremic effect is mediated via TSL-induced SIADH,^23^ with a median TTO of 29 days (IQR 13-84 days)-aligning with literature-reported SIADH-associated hyponatremia (after ~63 days, 3 treatment cycles).^24^

Psychotropic medications carry risk of inducing SIADH, which may cause hyponatremia.^25^ The FEARs database demonstrated predominant reporting counts for antidepressant agents, especially SSRIs that with strong correlation signals: citalopram 8.74 (95%CI 8.13-9.41), escitalopram 7.01 (95% CI 6.43-7.64), sertraline 5.54 (95% CI 5.19-5.92), fluoxetine 4.49 (95% CI 4.05-4.97). Our study showed oxcarbazepine had a significantly higher ROR (24.47, 95% CI 22.72-26.37), Consistent with prior studies, oxcarbazepine had the highest reporting frequency among AEDs in our pharmacovigilance data.^25^ FAERS mining showed antipsychotics (olanzapine, risperidone, quetiapine) were among the top 50 pharmacovigilance signals, but had lower RORs (1.39-3.18) vs. antidepressants (2.38-8.74) and antiepileptics (2.33-24.47). This lower risk quantification comes from prevalent psychotropic polypharmacy and confounding factors (compulsive water drinking/psychogenic polydipsia) in psychiatric patients.^26^

Furthermore, our study identified hyponatremia pharmacovigilance signals in drugs without such warnings on official labels, including CCBs, ARBs, bisphosphonates, metformin, and cytotoxic agents. A Swedish case-control study showed newly prescribed antihypertensives may moderately raise hyponatremia hospitalization risk.^8^ Bisphosphonate-induced hyponatremia has been sporadically reported with acute onset,^27^ while our data mining found prolonged TTO differences (zoledronic acid: n=146, median TTO 72.5 days; alendronate: n=76, median TTO 240.5 days), needing further clinical validation. Notably, zoledronic acid, pregabalin, rituximab, and lenalidomide ranked among the top 50 in reporting counts but had RORs <1. Our analysis suggests their large number of non-hyponatremia adverse event reports may have diluted hyponatremia signals, requiring continued surveillance of potential associations.

### Limitations:

While FAERS is a widely accepted pharmacovigilance system, it has limitations for druginduced hyponatremia analysis. First, FAERS adverse event reports need no proof of drug causality, and event details are unavailable-so hyponatremia occurrence cannot fully rule out underlying comorbidities. Second, partial data missing prevented exploring the impact of drug dosage and concomitant meds on hyponatremia. Third, limited data barred tracking patients’ serum Na changes, precluding accurate determination of the correlation between poor prognosis and hyponatremia. We recommend considering these limitations when interpreting findings; clinical judgment should integrate patients’ disease status and concomitant meds to assess hyponatremia-drug links.

## CONCLUSION

Data mining based on FAERS has validated the real-world association between hyponatremia and multiple medications, providing evidence for drug safety alerts, serving as a valuable reference for clinical decision-making, and supporting the optimization of medication safety strategies. This approach offers critical insights to enhance pharmacovigilance and patient care.
